# Serum metabolomic analysis in patients with Hashimoto’s thyroiditis

**DOI:** 10.3389/fendo.2022.1046159

**Published:** 2022-12-22

**Authors:** Xiao Jiang, Xinyu Zhao, Xiaotong Gu, Tao Luo, Pengqian Li, Chuchu Wan, Haixia Liu

**Affiliations:** ^1^ Endocrinology and Metabolism Department, The Second Affiliated Hospital of Dalian Medical University, Dalian, China; ^2^ Graduate School Department, Dalian Medical University, Dalian, China

**Keywords:** Hashimoto’s thyroiditis, metabolites, euthyroidism, subclinical hypothyroidism, ROC curve

## Abstract

**Background:**

Hashimoto’s thyroiditis, an autoimmune thyroid disease, shows high morbidity worldwide, particularly in female. Patients with Hashimoto’s thyroiditis have an increasing risk of hypothyroidism during the occurrence and progression of Hashimoto’s thyroiditis. In recent years, metabolomics has been widely applied in autoimmune diseases, especially thyroid disorders. However, metabolites analysis in Hashimoto’s thyroiditis is still absent.

**Methods:**

A total of 92 samples were collected, including 35 cases in the control group, 30 cases in the Hashimoto’s thyroiditis with euthyroidism group, and 27 cases in the Hashimoto’s thyroiditis with subclinical hypothyroidism group. SPSS 25.0 for statistical analysis and ROC curve, SIMCA 14.0, Metaboanalysis for multifactor analysis, and Origin 2021 for correlation analysis.

**Results:**

21 metabolites were identified. 10 metabolites were obtained from control group versus HTE group, 8 serum metabolites were abnormal between control group and HTS group, 3 metabolites were derived from HTE group versus HTS. Kyoto Encyclopedia of Genes and Genomes Enrichment analysis showed that fatty acid degradation, Arginine, and proline metabolism have a significant impact on HTE, while lysine degradation, tyrosine metabolism play an important role HTS group, compared to control group. In the comparison between the HTE and HTS group, Valine, leucine, and isoleucine degradation and Valine, leucine, and isoleucine biosynthesis exists a key role. Correlation analysis shows clinical are not related to metabolites. ROC curve indicates SM, LPC, PC can efficiency in identification patients with HT in different clinical stage from healthy individuals.

**Conclusion:**

Serum metabolites were changed in HT. Phospholipids such as SM, LPC, PC influence the pathogenesis of Hashimoto’s thyroiditis. Fatty acid degradation and lysine degradation pathways have an impact on different clinical stage of HT.

## Introduction

Hashimoto’s thyroiditis (HT), an autoimmune thyroid disease, shows high morbidity worldwide, particularly in female. Epidemiology shows that the prevalence of HT is about 10-17% (1, 2). Patients with HT have an increasing risk of hypothyroidism during the occurrence and progression of HT. Approximately 20% of patients develop various complications, including Addison’s disease, type 1 diabetes, pernicious anemia, and other autoimmune diseases (1, 3–5). Serum TPOAb or TgAb will be elevated in HT. However, HT progression is rarely predicted by hallmarks.

Metabolomics is a research approach to exploring how metabolites affect physiological and pathological in human by quantitative analysis of metabolites in human (6). Glucose, lipids, and amino acids are common metabolites in human.

Glucose is the major energy source for energy metabolism, Glucose metabolism pathways are strongly associated with HT. The mTOR/HIF-1a/HK2/glycolytic pathway of CD4^+^ T cells was activated in HT, while interrupted by 2DG and metformin, the function and differentiation of CD4^+^ T cells were affected 2DG and metformin served therapeutic significance (7).

Lipids can maintain the normal function of cell membranes, regulate energy metabolism, and engage in a significant biological pathway. HT is commonly associated with disorders of lipid metabolism. HT patients frequently accompany with dyslipidemia, and the blood lipid status of HT patients are ameliorated after L-thyroxine treatment (8). Jia Liu et al. analyzed small molecule metabolites in clinical hyper- and hypothyroidism and found abnormal serum levels of lipids and amino acids (phosphatidylcholine (PC), lysophosphatidylcholine (LPC), sphingomyelin (SM), glycodeoxycholic acid (GDCA), et al), with 22 metabolite changes in the hypothyroidism group and 17 metabolites in the hyperthyroidism group (9).

As the basic building blocks of peptides and proteins, amino acids perform a variety of physiological functions. Song et al. found that serum polyamine metabolite concentrations are different between patients with autoimmune thyroid disease and healthy controls. Decreased spermine: spermidine ratio in Graves Disease(GD), HT, and pTAb patients hypothesized that thyroid autoimmunity was associated with lower spermine levels (10).

Meantime, Chng et al. found that antithyroid drug treatment increased the arginine levels during the transition from hyperthyroidism to euthyroidism in GD patients (11). Xing Zhang et al. found that metabolic network and inflammatory factors were influenced and cured by Yanghe decoction in HT (12).

Nevertheless, metabolites in the progression of HT are still absent. Therefore, our study aimed to screen for metabolites that could be used to predict progression by assessing abnormalities in serum metabolites at different clinical stages of HT, as well as the correlation of metabolites with thyroid antibodies.

## Method

### Samples

The study protocol was approved by the Ethics Committee of the Second Hospital of Dalian Medical University (approval number 2021 NO.073). All studies were conducted in accordance with the principles of the Declaration of Helsinki. Ninety-two samples were included and divided into three groups in our research. Fifty-seven patients with HT attending the Department of Endocrinology at Dalian Medical University were included in the experimental group, of which 30 were included in the HT with euthyroidism group and 27 were included in the HT with subclinical hypothyroidism group. Thirty-five healthy individuals were collected from the medical examination center of the Second Hospital of Dalian Medical University and assigned to the control group. The inclusion and exclusion criteria were as follows.

### Inclusion criteria

HT with euthyroidism group fulfills (a) Diffuse swelling of the thyroid gland with surface roughness and nodularity by ultrasound. (b) Anti-thyroglobulin antibodies (TgAb) and/or thyroid peroxidase antibodies (TPOAb) are positive. (c) The serum FT3, FT4, and thyroid stimulating hormone (TSH) levels are normal. (d)Without any drugs or medicine.

HT with subclinical hypothyroidism group fulfills, (a) Diffuse swelling of the thyroid gland with surface roughness or nodularity by ultrasound. (b) Anti-thyroglobulin antibodies (TgAb) and thyroid peroxidase antibody (TPOAb) positive. (c)The serum thyroid stimulating hormone (TSH) level increased, FT3, FT4 levels are normal. (d)Without any drugs or medicine.

### Exclusion criteria

All group that fulfills any criteria will be excluded, (a) Patients who also have other autoimmune diseases, such as diabetes mellitus, lupus, and inflammatory bowel disease, etc. (b) Patients complicated with concurrent acute and chronic infectious diseases, such as acute and chronic hepatitis, pneumonia, etc. (c) Patients who are taking non-steroidal drugs and glucocorticoid drugs at the same time. (d) Patients were complicated with concurrent malignant tumors or immune function deficiency. (e) Pregnant or lactating patients.

### Metabolic profiling -LC-MS full-component data acquisition

Metabolites were detected using the LC-MS full-component data system. Chromatographic separations were performed in positive and negative ion modes.

#### Positive ion mode

##### Chromatographic column

Waters BEH C8 column (size: 50 mm × 2.1 mm, 1.7 μm) (Waters, Milford, MA), column temperature: 60°C, flow rate: 0.4ml/min.Mobile phases: water with 0.1% formic acid (phase A) and acetonitrile with 0.1% formic acid (phase B).Gradient: The starting gradient was 5% B, maintained for 0.5 min, followed by a linear increase to 40% B within 1.5 min, and another linear increase to 100% B within 6 min and maintained for 2 min, and then decreased back to the initial gradient of 5% B at 10.1 min and equilibrated for 2 min.

#### Negative ion mode

##### Chromatographic column

ACQUITY UPLC HSS T_3_ (size: 50 mm × 2.1 mm, 1.8 μm) (Waters, Milford, MA), column temperature: 60°C, flow rate: 0.4 ml/min.Mobile phase: Phase A was water plus 6.5 mM NH4HCO3; Phase B was aqueous solution containing 95% methanol and 6.5 mM NH4HCO3.Gradient: The starting gradient was 2% B, maintained for 0.5 min, increased to 40% B within 2 min, then increased linearly to 100% B within 6 min and maintained for 2 min, and decreased back to the initial gradient of 2% B at 10.1 min with an equilibrium of 1.9 min.

### Mass spectrometry data acquisition parameters

Mass spectrometry (MS) full scan range positive ions m/z 80-1200, spray voltage 3.50 kV; negative ions 80-1200, spray voltage 3.00 kV. capillary temperature 300°C, auxiliary heating gas temperature 350°C, sheath gas and auxiliary gas flow rate 45 and 10 (arbitrary units), respectively. The resolution was set to 7e4. Internal standard for serum sample analysis was shown in **Table S1**.

### Statistical analysis

The normally distributed data from Clinical parameters of the control, hyperthyroidism, and hypothyroidism groups were expressed by mean ± standard deviation. Variables with skewed distributions are described with 95% confidence intervals. The differences among the three groups were analyzed by analysis of t-tests or non-parametric tests.

All statistical analyses were performed by Statistical Package for the Social Sciences (SPSS) 25.0 (SPSS, Chicago, IL, USA), and the results were considered statistically significant with two-tailed analyses, P< 0.05. Partial least squares discriminant analysis(PLS-DA) and Orthogonal projections to latent structures discriminant analysis(OPLS-DA) were carried out by SIMCA 14.1 (Umetrics AB, Umea, Sweden). Serum metabolites with VIP >1.5 in the OPLS-DA model were assessed for statistical significance by t-test or non-parametric test.

Data were normalized using MetaboAnalyst 5.0 (https://www.metaboanalyst.ca/) to reduce systematic bias and improve consistency. Remove features with >25% missing values and the remaining missing values were replaced by the mean in the original data. Normalized by sum and mean-centered and divided by the square root of the standard deviation of each variable. Enrichment analysis and heatmap of metabolite were conducted by MetaboAnalyst.

Correlation analysis was performed by Origin 2021.The ROC curve was established by SPSS. ROC curves for differential metabolites were plotted by SPSS to determine diagnostic efficiency. Metabolite point plotting was drawn by Graph prism 9.0.

## Results

### Baseline parameters

The baseline parameters of the control, HT with euthyroidism (HTE) group were shown in **Table 1**. There was a significant difference in thyrotropin (TSH), free triiodothyronine (FT_3_), free thyroxine (FT_4_), thyroglobulin antibody (TgAb), thyroid peroxidase antibody (TPOAb), total cholesterol (TC), triglyceride (TG), high-density lipoprotein cholesterol (HDL), low-density lipoprotein cholesterol (LDL), fasting plasma glucose (FPG) between the two groups (p<0.05). The levels of TSH, TgAb, TPOAb, TC, TG, HDL and LDL were increased in HT with euthyroidism group while FT_3_, FT_4_ and FPG were decreased.

**Table 1:** The clinical baseline parameters of the control and HTE group.

Control vs HT with Euthyroidism			
Parameters	Control n=35	HT with Euthyroidism n=30	P
Sex, M/F, n	13/22	1/29	
Age, y	37.80 ± 1.78	43.13 ± 2.32	
TSH, mIU/L	1.67 ± 0.84	2.24 ± 1.02	0.016*
FT_3_, pmol/L	5.12 ± 0.48	4.85 ± 0.44	0.02*
FT_4_, pmol/L	15.85 ± 0.35	15.21(14.3-16.1,95%CI)	0.214
TgAb, U/ml	16.31 (14.3-18.28,95%CI)	222.31 ± 27.87	<0.001*
TPOAb, U/ml	31.01 (29.02-32.99,95%CI)	897.01(700.91-1093.10,95%CI)	<0.001*
TC, mmol/L	4.11 (3.89-4.33,95%CI)	5.24(5.12-5.36,95%CI)	<0.001*
TG, mmol/L	1.19 ± 0.063	1.29(1.20-1.38,95%CI)	0.032*
HDL,mmol/L	1.29 (1.23-1.35,95%CI)	1.54(1.50-1.57,95%CI)	<0.001*
LDL, mmol/L	2.44 (2.29-2.58,95%CI)	3.11(3.06-3.16,95%CI)	<0.001*
FPG, mmol/L	5.40 (5.27-5.52,95%CI)	5.25(5.19-5.31,95%CI)	0.006*

*represent P<0.05.

Normal Ranges.

TSH,mIU/L 0.38-4.34 FT_3_, pmol/L 2.77-6.31.

FT_4_,pmol/L 10.44-24.38 TgAb,U/ml 0-60.

TPOAb,U/ml 0-60 FPG,mmol/L 3.9-6.1.

TC, mmol/L 2.9-5.17 TG,mmol/L 0.22-1.7.

HDL,mmol/L 0.9-2.19 LDL,mmol/L 0-3.36.

The clinical baseline data of the control group and HT subclinical hypothyroidism group (HTS) are listed in **Table 2**. There was significance in both groups in TSH, FT_4_, TgAb, TPOAb, TC, TG, and HDL (p<0.05). There was no statistical significance in FT_3_, LDL, and FPG. TSH, TgAb, TPOAb, TC, and FT_3_ were elevated in the HTS group, while TG, LDL and FT_4_ were reduced.

**Table 2:** The clinical baseline parameters of the control and HTS group.

Control vs HT with Subclinical Hypothyroidism			
Parameters	Control n=35	HT with Subclinical Hypothyroidism n=27	P
Sex, M/F, n	13/22	3/24	
Age, y	37.80 ± 1.78	36.48 ± 1.981	
TSH, mIU/L	1.67 ± 0.84	7.58 (6.32-8.85,95%CI)	<0.001*
FT_3_, pmol/L	5.12 ± 0.48	5.31 (4.49-6.13,95%CI)	0.284
FT_4_, pmol/L	15.85 ± 0.35	13.72 ± 0.52	0.001*
TgAb,U/ml	16.31 (14.3-18.28,95%CI)	281.57 (214.80-348.34,95%CI)	<0.001*
TPOAb, U/ml	31.01 (29.02-32.99,95%CI)	1046.50 (867.91-1225.08,95%CI)	<0.001*
TC, mmol/L	4.11 (3.89-4.33,95%CI)	4.70 (4.56-4.83,95%CI)	<0.001*
TG, mmol/L	1.19 ± 0.063	0.97 (0.91-1.03,95%CI)	<0.001*
HDL, mmol/L	1.29 (1.23-1.35,95%CI)	1.53 (1.45-1.61,95%CI)	<0.001*
LDL, mmol/L	2.44 (2.29-2.58,95%CI)	2.41 (2.30-2.52,95%CI)	0.129
FPG, mmol/L	5.40(5.27-5.52,95%CI)	5.28 (5.22-5.34,95%CI)	0.017*

*represent P<0.05.

Normal Ranges.

TSH,mIU/L 0.38-4.34 FT_3_, pmol/L 2.77-6.31.

FT_4_,pmol/L 10.44-24.38 TgAb,U/ml 0-60.

TPOAb,U/ml 0-60 FPG,mmol/L 3.9-6.1.

TC, mmol/L 2.9-5.17 TG,mmol/L 0.22-1.7.

HDL,mmol/L 0.9-2.19 LDL,mmol/L 0-3.36.

The baseline parameters of the HTE group and HTS group were presented in **Table 3**. TSH, TC, TG, HDL, LDL, and FPG have a significant difference(p<0.05). The concentration of TSH, FT_3_, TgAb, TPOAb and FPG was higher in HT with the subclinical hypothyroidism group while TC, TG, HDL and LDL were lower. FT_3_, FT_4_, TgAb, and TPOAb were shown no significant difference.

**Table 3:** The clinical baseline parameters of the HTE and HTS group.

HT with Euthyroidism vs HT with Subclinical Hypothyroidism			
Parameters	HT with Euthyroidism n=30	HT with Subclinical Hypothyroidism n=27	P
SexT_3_, M/F, n	1/29	3/24	
Age, y	43.13 ± 2.32	36.48 ± 1.981	
TSH, mIU/L	2.24 ± 1.02	7.58 (6.32-8.85,95%CI)	<0.001^*^
FT_3_, pmol/L	4.85 ± 0.44	5.31 (4.49-6.13,95%CI)	0.306
FT_4_, pmol/L	15.21 (14.3-16.1,95%CI)	13.72 ± 0.52	0.057
TgAb, U/ml	222.31 ± 27.87	281.57 (214.80-348.34,95%CI)	0.183
TPOAb, U/ml	897.01 (700.91-1093.10,95%CI)	1046.50 (867.91-1225.08,95%CI)	0.358
TC, mmol/L	5.24 (5.12-5.36,95%CI)	4.70 (4.56-4.83,95%CI)	<0.001^*^
TG, mmol/L	1.29 (1.20-1.38,95%CI)	0.97 (0.91-1.03,95%CI)	<0.001^*^
HDL, mmol/L	1.54 (1.50-1.57,95%CI)	1.53 (1.45-1.61,95%CI)	<0.001^*^
LDL, mmol/L	3.11 (3.06-3.16,95%CI)	2.41 (2.30-2.52,95%CI)	<0.001^*^
FPG, mmol/L	5.25 (5.19-5.31,95%CI)	5.28 (5.22-5.34,95%CI)	<0.001^*^

*represent P<0.05.

Normal Ranges.

TSH,mIU/L 0.38-4.34 FT3, pmol/L 2.77-6.31.

FT_4_,pmol/L 10.44-24.38 TgAb,U/ml 0-60.

TPOAb,U/ml 0-60 FPG,mmol/L 3.9-6.1.

TC, mmol/L 2.9-5.17 TG,mmol/L 0.22-1.7.

HDL,mmol/L 0.9-2.19 LDL,mmol/L 0-3.36.

### Metabolomic analysis

**Figure 1:**
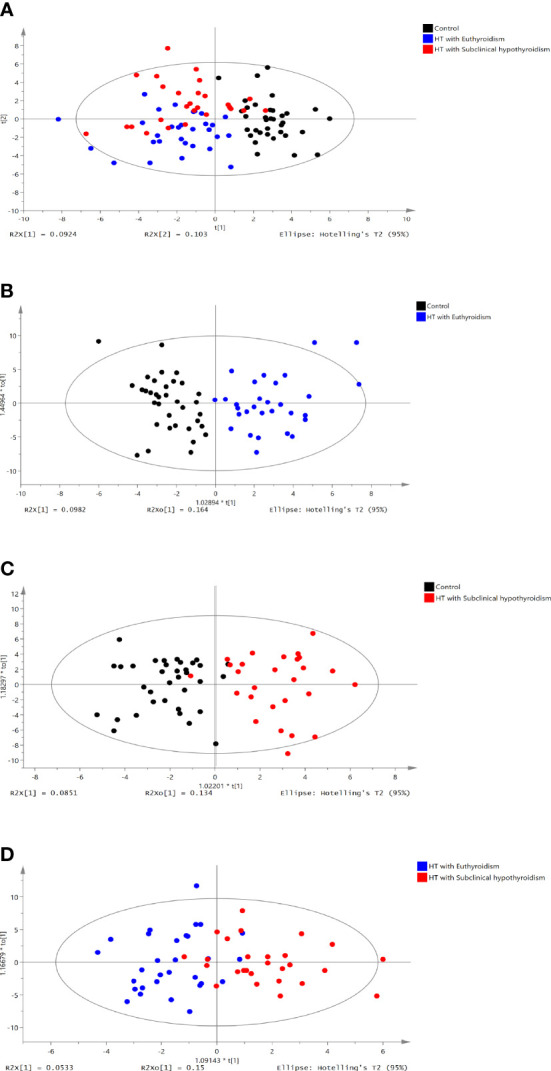
PLS-DA score plot of the control vs HT with euthyroidism,HT with subclinical hypothyroidism groups. **(A)** PLS-DA score plot of three groups (R2X =0.195, R2Y = 0.44, Q2 = 0.281). **(B)** OPLS-DA score plot of the control group vs. the HT with euthyroidism group (R2X =0.262, R2Y = 0.756, Q2 = 0.641). **(C)** OPLS-DA score plot of the control vs HT with subclinical hypothyroidism group (R2X=0.203, R2Y=0.56, Q2 = 0.233). **(D)** OPLS-DA score plot of HT with euthyroidism group vs. HT with subclinical hypothyroidism group (R2X =0.203, R2Y = 0.563, Q2 = 0.233). The t [1] and t [2] values in the figures represent the scores of each sample in principal components 1 and 2, respectively. Each dot on the plot represents a sample in the corresponding group.

The 219 metabolites were assayed among three groups (**Table S2**). Partial least squares discriminant analysis (PLS-DA) was used to distinguish three groups (R2X =0.195, R2Y = 0.44, Q2 = 0.281, **Figure 1A**). The Control group (black dots) and HTE (blue dots) group were significantly separated *via* Orthogonal projections to latent structures discriminant analysis (OPLS-DA) (R2X =0.262, R2Y = 0.756, Q2 = 0.641, **Figure 1B**). The control (black dots) group and HTS group with subclinical hypothyroidism (red dots) groups were significantly separated *via* OPLS-DA (R2X=0.219, R2Y=0.718, Q2 = 0.570, **Figure 1C**). THE (blue dots) and HTS (red dots) group was divided *via* OPLS-DA analysis (R2X =0.203, R2Y = 0.563, Q2 = 0.233, **Figure 1D**). On account of the screening criteria of variable influences on projection (VIP)>1.5 and P<0.05,21 metabolites were identified. 10 metabolites were obtained from control group vs HTE group, 8 metabolites were selected from HTE group vs HTS group, 3 metabolites were derived from HTS group vs HTE group (**Tables 4.1**, **4.2**).

**Table 4.1:** The identification metabolites of control vs HTE and control vs HTS group.

Control vs HT with Euthyroidism			Control vs HT with Subclinical Hypothyroidism		
Metabolites	VIP	P	Metabolites	VIP	P
PC 38:6	<0.001	6.12897	SM 34:1	<0.001	6.15548
SM 34:1	<0.001	5.66715	LPC 18:0 sn-1	<0.001	5.06263
LPC 18:2 sn-1	0.030	4.81601	PC 38:6	0.002	4.89183
LPC 18:0 sn-1	<0.001	3.28948	SM 34:2	0.001	3.07559
LPC 18:1 sn-1	<0.001	3.03826	LPC 18:1 sn-1	<0.001	2.50319
SM 34:2	<0.001	2.65928	SM 36:2	0.038	2.43413
PC 36:5	0.013	2.52393	Hexadecenoylcarnitine	0.03	2.03925
PC 34:3	<0.001	2.00065	PC 36:3	0.022	1.55211
L-Acetylcarnitine	0.022	1.95776			
SM 36:2	<0.001	1.92598			

**Table 4.2:** The identification metabolites of HTE vs HTS group.

HT with Euthyroidism vs HT with Subclinical Hypothyroidism		
Metabolites	P	VIP
LPC 18:0 sn-1	0.031	3.98814
FFA 18:0	0.030	3.75871
PC 32:2	0.007	1.81145

**Figure 2:**
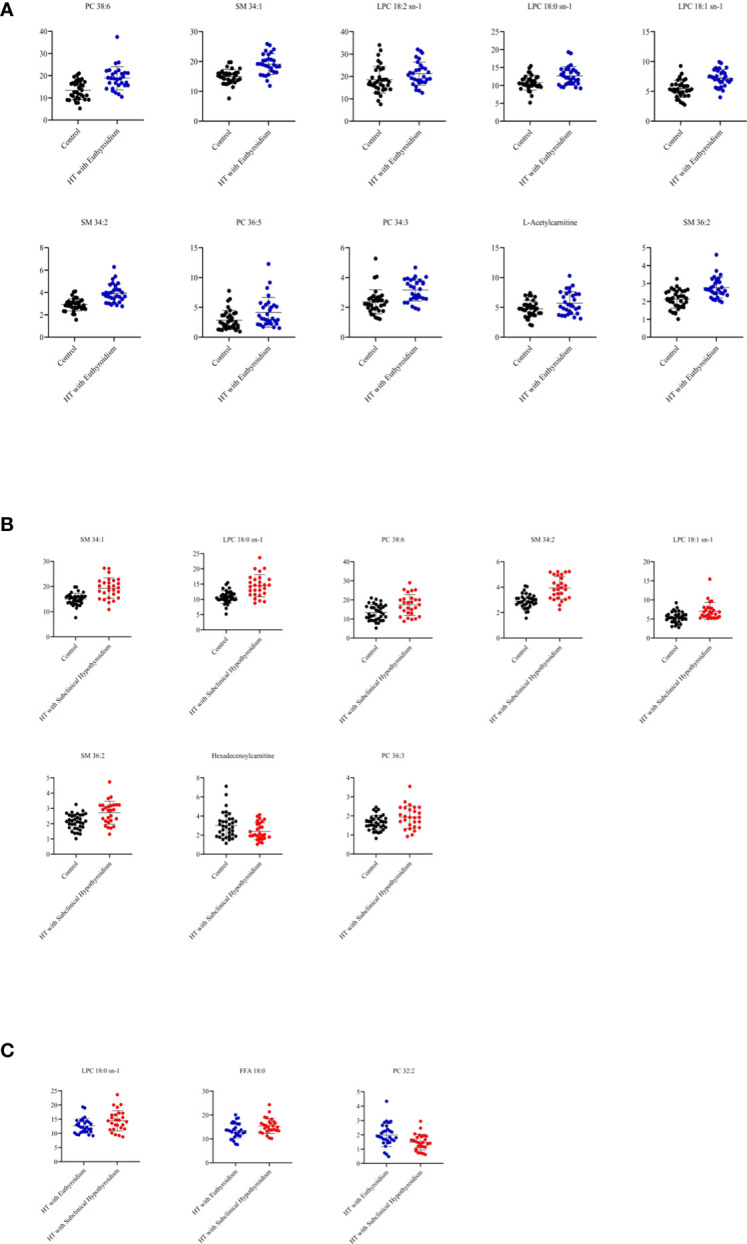
**(A)** The metabolites from comparison between the control and HT with euthyroidism group. **(B)** The metabolites from comparison between the control and HT with subclinical hypothyroidism group. **(C)** The metabolites from comparison between the HT with euthyroidism and HT with subclinical hypothyroidism group.

**Figure 3:**
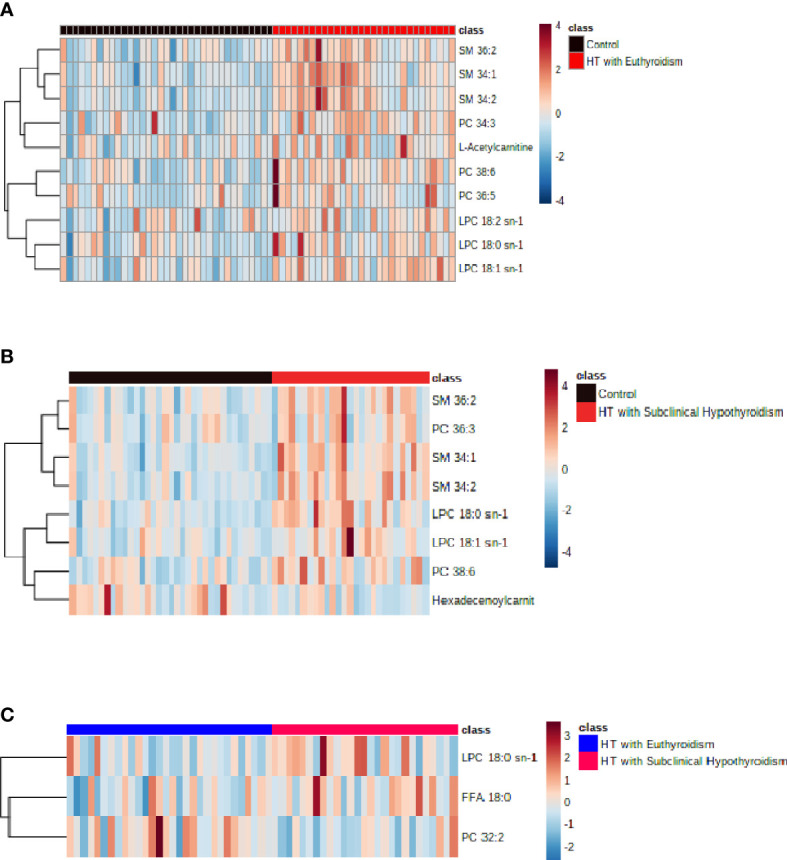
**(A)** The heatmap of metabolites from comparison between the control and HT with euthyroidism group. **(B)** The heatmap of metabolites from comparison between the control and HT with subclinical hypothyroidism group.**(C)** The heatmap of metabolites from comparison between the HT with euthyroidism and HT with subclinical hypothyroidism group.

In the comparison between the control group and HTE group, phosphatidylcholine (PC) 38:6, sphingomyelin (SM) 34:1, lysophosphatidylcholine (LPC) 18:2 sn-1, LPC 18:0 sn-1, LPC 18:1 sn-1, SM 34:2, PC 36:5, PC 34:3, L-Acetylcarnitine and SM 36:2 were higher in HT with euthyroidism patients than healthy (**Figure 2A**). The heatmap of metabolites was shown in **Figure 3A**.

Compared to the control group, the HTS group has a higher concentration of SM 34:1, LPC 18:0 sn-1, PC 38:6, SM 34:2, LPC 18:1 sn-1, SM 36:2, PC 36:3, while Hexadecenoylcarnitine was lower (**Figure 2B**). The heatmap of metabolites presented the number of metabolites (**Figure 3B**). The level of LPC 18:0 sn-1 and free fatty acid (FFA) 18:0 were increased in HT with subclinical hypothyroidism than in HT with euthyroidism while PC 32:2 was decreased (**Figure 2C**). The selected metabolites of two groups were distributed in the heatmap (**Figure 3C**).

Both HT patients have increased concentration of LPC 18:0 sn-1 which was higher in HT with subclinical hypothyroidism.

### Correlation analysis

**Figure 4:**
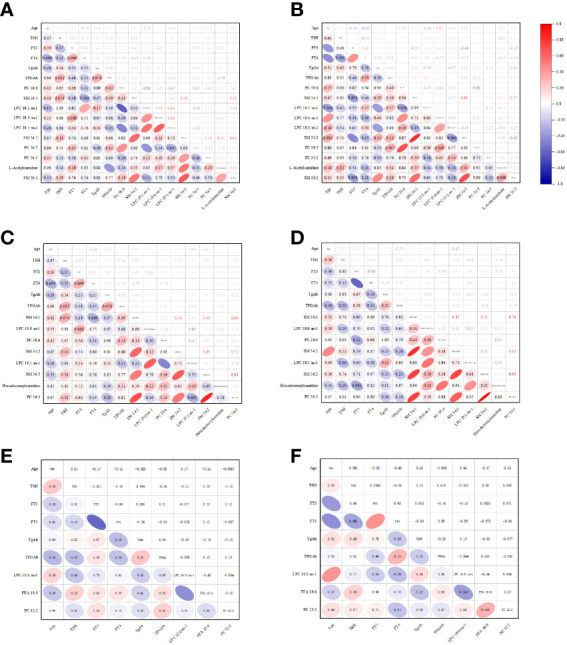
The Pearson correlation coefficients between the clinical variables and the metabolites were displayed by Correlograms. **(A)** The analysis between the clinical variables of control and the metabolites of control vs HTE. **(B)** The analysis between the clinical variables of HTE and the metabolites of control vs HTE. **(C)** The analysis between the clinical variables of control and the metabolites of control vs HTS. **(D)** The analysis between the clinical variables of HTS and the metabolites of control vs HTS. **(E)** The analysis between the clinical variables of HTE and the metabolites of HTE vs HTS. **(F)** The analysis between the clinical variables of HTS and the metabolites of HTE vs HTS.

The correlation analysis of clinical data and metabolites was carried out by Origin 2021. The Pearson correlation coefficients between the clinical variables and the metabolites were displayed by Correlograms with different colors (**Figures 4A–F**).

No correlation or weak correlation was presented in the correlation analysis between clinical data and metabolites among three groups (Pearson correlation coefficient >0.7, P<0.05).

SM 34:1 and SM 34:2 was shown strong correlation in HTE group.

### Enrichment analysis

Enrichment analysis was performed to confirm the significantly changed metabolic pathway based on the Kyoto Encyclopedia of Genes and Genomes database through Metaboanalyst.

**Figure 5:**
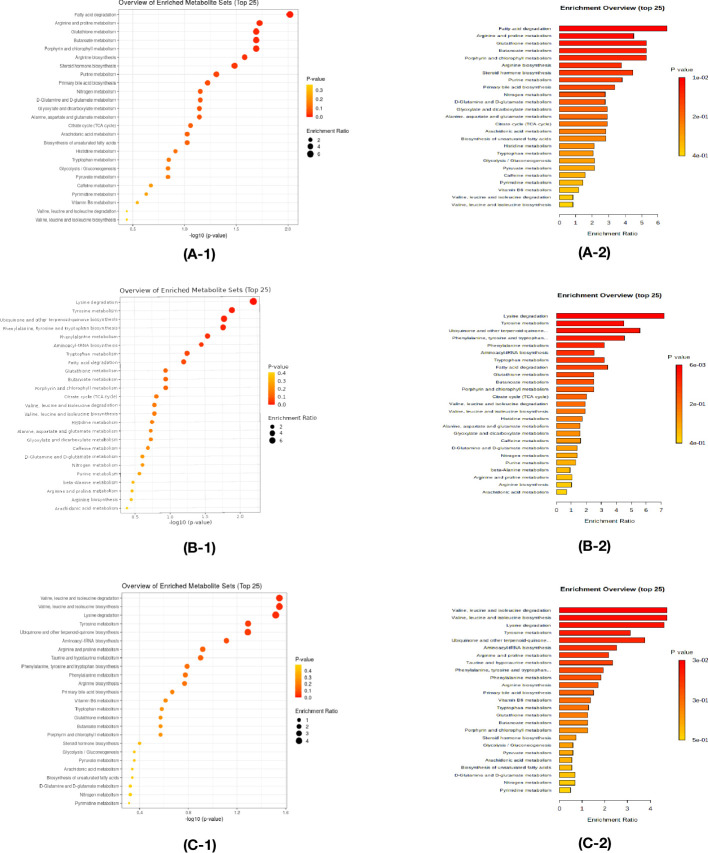
Enrichment analysis was performed to confirm the significantly changed metabolic pathway based on the Kyoto Encyclopedia of Genes and Genomes database through Metaboanalyst. (A-1,A-2) The comparison between the control and HT with euthyroidism group. (B-1,B-2) The comparison between the control and HT with subclinical hypothyroidism group. (C-1,C-2) The comparison between the HT with euthyroidism and HT with subclinical hypothyroidism group.

In the comparison between the control and HTE group, Fatty acid degradation, Arginine, and proline metabolism have a significant impact (**Figures 5A1, A2**). Lysine degradation, Tyrosine metabolism play an important role (**Figures 5B1, B2**) in the comparison between the control and HTS group. In the comparison between the HTE and HTS group, Valine, leucine, and isoleucine degradation and Valine, leucine, and isoleucine biosynthesis exists a key role (**Figures 5C1, C2**).

### ROC curve

**Figure 6:**
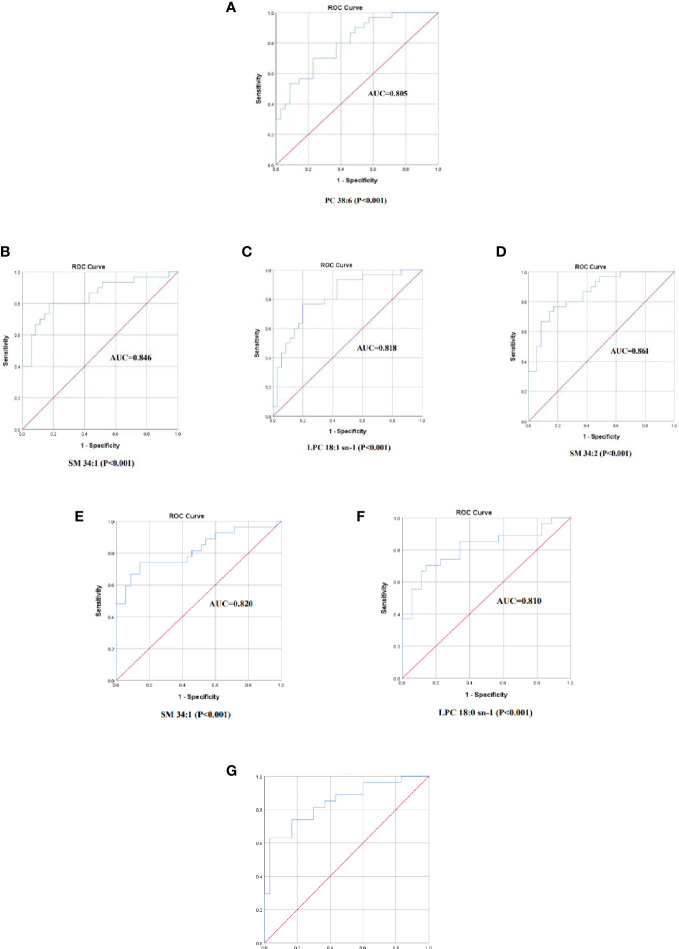
The ROC curve of selected metabolomics. **(A)** PC 38:6, AUC=0.805. **(B)** SM 34:1, AUC=0.846. **(C)**LPC 18:1 sn-1, AUC=0.818. **(D)** SM34:2, AUC=0.861. **(E)** SM 34:1, AUC=0.820. **(F)** LPC 18:0 sn-1, AUC=0.810. **(G)** X=0.379(LPC 18:0 sn-1)+0.395(FFA 18:0)-1.537(PC 32:2)-8.246,AUC =0.843, 95%.

The screened differential metabolites have seemed as potential biomarkers for the area under the ROC curve analysis. SM 34:1 was the most efficient in distinguishing the Control group from the HTE group (**Figure 6B**, AUC=0.846, 95% CI: 0.747-0.944). The AUCs of LPC 18:1 sn-1, PC 38:6, and SM34:2 were also greater than 0.8 (**Figures 6A, C, D**).

SM 34:1 was also found to be the most efficient in discriminating the Control group from the HTS group (**Figure 6E**, AUC=0.820, 95% CI: 0.708-0.932). The AUC of LPC 18:1 sn-1 was also greater than 0.8 (**Figure 6F**).

However, AUC curves for 3 screened differential metabolites were less than 0.8 in differentiating the HTE group from the HTS group. The prediction model was performed by a binary regression:


X=0.379(LPC 18:0 sn−1)+0.395(FFA 18:0)−1.537(PC 32:2)−8.246

The AUC =0.843, 95% CI: 0.740-0.946 with the ROC curve area analysis. THE group and HTS are effectively identified by this prediction model (**Figure 6G**).

## Discussion

Our study found differences in serum metabolites among patients with HTE, patients with HTS, and healthy individuals. 10 metabolites were abnormal in the comparison between the control and HTE group. Phosphatidylcholine (PC) 38:6, sphingomyelin (SM) 34:1, lysophosphatidylcholine (LPC) 18:2 sn-1, LPC 18:0 sn-1, LPC 18:1 sn-1, SM 34:2, PC 36:5, PC 34:3, L-Acetylcarnitine and SM 36:2 were higher in HTE. 8 metabolites that fulfilled the criterion were selected from the control and HTS group. The level of SM 34:1, LPC 18:0 sn-1, PC 38:6, SM 34:2, LPC 18:1 sn-1, SM 36:2, and PC 36:3 were increased in HTS, whereas Hexadecenoylcarnitine was decreasing. In compare to HTE and HTS groups, HTS serum contains more LPC 18:0 sn-1 and free fatty acid (FFA) 18:0, less PC 32:2.

Sphingomyelin can mediate cellular stress and inflammation (13). Increased pro-inflammatory cytokines and chemokines can induce HT (1). IFN-γ and tumor necrosis factor (TNF)-α produced by Th1 lymphocytes trigger factors released by thyroid cells. Then thyroid autoimmunity happened. IFN-γ and tumor necrosis factor (TNF)-α promote the release of sphingomyelin hydrolase. In turn, sphingomyelin triggers inflammatory cytokine synthesis and release (14). Sphingomyelin interacts with the development of HT.

Phospholipid formation and metabolism are closely related to the function of immune cells (15). The imbalance of helper T (Th)/regulatory T (Treg) cells is a key of the pathogenic mechanisms of HT. Then, abnormal activation of CD4^+^ T cells attacks thyroid cells (1). The uptake of ATP by activated CD4^+^ T cells depends on fatty acid synthesis, glycolysis and glutaminolysis, leading to differentiation towards Th cells (16). Previous studies have shown that the activation of healthy peripheral blood CD4^+^ T cells promotes the involvement of partial fatty acids in lipid synthesis, phospholipid synthesis, lipoprotein translation and modification processes (17). The concentrations of phospholipid increased in patients with HT at different clinical stages and in different categories. the potential causes is the abnormal activation of CD4^+^ T cells leading to increased phospholipid levels and the ratio of Th cells.

Lysophospholipids are a class of signaling molecules that have immune regulatory functions. Lysophospholipids released by apoptotic cells recruit monocytes and lymphocytes (18, 19), causing the clearance of apoptotic cell debris and limiting the activation of the immune system by secondary necrosis (20).

PC is elevated in the serum of patients who suffer HT at different clinical stages. It may be involved in the occurrence and development of HT by modulating inflammatory factors and altering immune cell function and action. The area under the ROC curve SM 34:1, LPC18:1 sn-1, and other PC were greater than 08. PC is an effect in distinguished HT from healthy (ROC>0.8) and is probably one of the potential markers of HT in the early stage.

Fatty acids are compounds composed of carbon, hydrogen and oxygen, which are the main components of neutral fats, phospholipids and glycolipids. Thyroid hormones, the main source of free fatty acids in humans, stimulate lipolysis in adipose tissue (21), while thyroid hormones promote the uptake of free fatty acids in peripheral tissues (22). According to our research, in comparison to HT with euthyroidism, free fatty acid (FFA) 18:0 was elevated in comparison to HT with subclinical hypothyroidism group. In contrast, FFA made no difference in the control group compared with the HT with euthyroidism group. Thyrotropin (TSH) was elevated and free thyroxine (FT_4_) was declined in HT patients with subclinical hypothyroidism. Decreased thyroid hormone will influence lipid metabolism, reduce fatty acid oxidation (FAO), decrease lipolysis in white adipose tissue and uptake of free fatty acid (23). The levels of fatty acids will be increased by thyroid hormone.

Carnitine is an antagonist of TH (24), mainly generated by incomplete fatty acid β-oxidation in mitochondria (25), which associated with the function of mitochondria. Studies have revealed that fatty acid β-oxidation is accelerated in the mitochondria of activated CD4^+^T cells (16). The mitochondria of CD4^+^T cells in Hashimoto’s disease are likely dysfunctional (7). The altered carnitine in patients with Hashimoto’s disease may be related to mitochondria.

Kyoto Encyclopedia of Genes and Genomes (KEGG) pathway enrichment analysis is carried out. Compared to the control group, HTE is mainly influenced by fatty acid degradation, HTS by Lysine degradation. For patients with HT, valine, leucine, and isoleucine degradation have a crucial effect on the HTE.

Fatty acid degradation, or fatty acid βoxidation, is mainly involved in the development and differentiation of Treg cells. Inhibitors of FAO rate-limiting enzyme carnitine palmitoyltransferase (CPT1A) blocked mitochondrial transport of long-chain fatty acids and inhibited Treg growth and differentiation (26). In a study of systemic sclerosis (SSc), AOttriad et al. found that incubation with etoposide, a carnitine transporter inhibitor, restrained fatty acid oxidation and suppressed the production of pro-inflammatory cytokines such as IL-6, revealing an altered metabolic state of the immune system in SSc patients and opening up new avenues of a potential treatment for the alleviation of inflammation (27). However, studies of fatty acid oxidation in HT are still lack, we assume that fatty acid B oxidation stimulates the release of factors of inflammation and modifies the function and differentiation of T cells, thus leading to the development of HT.

The disruption of lysine degradation affects the occurrence of HTS. Lysine is increased in the serum of HT patients, including pTAb, HTS, and HT (9). Lu X et al. found that tri-methylated histone H3 lysine 4 (H3K4me3), a marker of gene activation, was interrelated with genes of HT (28). As HT progresses in patients, the abnormalities of lysine degradation are more severe. There was no correlation between metabolites and patients’ age, gender, and thyroid parameters (FT3, FT4, TSH, TPOAb, TgAb) through correlation analysis. However, our study does not contains any polyamine metabolic, spermine was negatively correlated with thyroid-specific antibodies in research of the serum polyamine metabolic profile in HT (9).

Research on autoimmune diseases and metabolomics has been extensively advanced. In systemic lupus erythematosus (SLE), more than 200 metabolites (containing peptides, fatty acids, nucleotides, and carbohydrates) in serum were analyzed by liquid chromatography-spectroscopy (mass spectrometry), and all relevant metabolites were decreased (29). In patients with rheumatoid arthritis (RA), serum metabolomic profiles showed abnormalities in small molecule metabolites such as glucose, lactate, citrate, cholesterol, glycerol, and ribose compared to normal subjects, presumably as potential biomarkers for the diagnosis of RA (30).Metabolomic analysis of patients with type 1 diabetes found elevated levels of a group of small molecule metabolic markers (low-carbon number saturated lipids, lysophosphatidic- ethanolamine, and lysophosphatidylcholine) compared to normal subjects, implicating an association between metabolites and type 1 diabetes (31). A recent study of 30 patients with Graves’ disease (GD) showed that antithyroid treatment increased levels of LPC and SM (32).

In SLE, Amir Sharabi et al. revealed that medicines for SLE affect the metabolism of T-cell subsets (including glycolysis, glutaminolysis, fatty acid, and glycosphingolipid metabolism), altering metabolite levels (33). Xing Zhang et al. suggested that The levels of inflammatory factors and serum metabolites in mice with HT were changed after administration of Yanghe Decoctiont, indicating that Yanghe_Decoction reduces inflammatory factors in HT (12), while the metabolomic studies of HT after treatment are still absent in clinical.Consequently, we analyzed the serum metabolites of patients with HT in different clinical stages. LPC 18:0 sn-1, PC 38:6, SM 34:2, LPC 18:1 sn-1, and SM 36:2 were increased in both HTE and HTE groups compared to the control. HTE has a higher LPC 18:0 sn-1 than HTS. We suggested LPC 18:0 sn-1 is a risk factor to influence Hashimoto’s progress. Pathway enrichment analysis showed that fatty acid degradation and lysine degradation pathways play a central role in disease progression.

## Conclusion

In the early stages of HT, metabolites in the serum are abnormal. Metabolites will be further altered with the development of HT. Phospholipids such as SM, LPC, and PC influence the pathogenesis of HT. Fatty acid degradation and lysine degradation pathways play a central role in different clinical stages. Our study contributes to a further understanding of how metabolite levels and metabolism change in different clinical stages influence HT. However, longitudinal studies involving larger populations are needed to confirm these results, and further studies are necessary to explain the underlying mechanisms.

## Limitations

There are several limitations of our study. First, the sample size of our research is small. Second, metabolomics of HT patients after treatment is lack in our research. Besides, though correlation analysis shows no relation,we also are failure to match the age and sex among three group. In addition, we did not collect thyroid tissue or urine samples. Thus, we will conduct metabolomic researches of HT after clinical treatment in the future.

## Data availability statement

The original contributions presented in the study are included in the article/**Supplementary Material**. Further inquiries can be directed to the corresponding author.

## Ethics statement

The study protocol was approved by the Ethics Committee of the Second Hospital of Dalian Medical University (approval number:2021NO.073). Written informed consent for participation was not required for this study in accordance with the national legislation and the institutional requirements.

## Author contributions

Conceived and designed the experiments: XJ and XZ; Constructed figure and table: XG, PL, CW; Analyzed the data: TL; Wrote and translated: XJ and XZ. All authors contributed to the article and approved the submitted version.
