# Supplementary material for: Serum metabolomic analysis in patients with Hashimoto’s thyroiditis
Source: Front Endocrinol (Lausanne). 2022 Dec 22;13:1046159. doi: 10.3389/fendo.2022.1046159 (PMC9814722; doi:10.3389/fendo.2022.1046159)
Supplement: Supplementary file 1 [file DataSheet_1.docx]

**Table S1**

**Internal standard for serum sample analysis**

| **Name English** | **Abbreviation** | **Final concentration (ug/ml)** |
| --- | --- | --- |
| Acetyl carnitine-d3 | Carnitine C2:0-d3 | 0.1 |
| Decanoyl carnitine-d3 | Carnitine C10:0-d3 | 0.1 |
| Palmitoyl carnitine-d3 | Carnitine C16:0-d3 | 0.15 |
| Lysophosphatidylcholine | LPC 19:0 | 0.75 |
| Palmitic acid-d3 | FFA C16:0-d3 | 2.5 |
| Stearic acid-d3 | FFA C18:0-d3 | 2.5 |
| Tryptophan-d5 | Trp-d5 | 4.25 |
| Phenylalanine-d5 | Phe-d5 | 3.6 |
| Cholic acid-d4-d4 | CA-d4 | 1.85 |
| Goose deoxycholic acid-d4 | CDCA-d4 | 1.5 |

**Table S2**

| **Metabolites** | | | |
| --- | --- | --- | --- |
| Choline | Carnitine C12_0 | LPE 20:5 sn-2 | LPC 22_0 |
| leucine | Cortisone | LPE 20:5 sn-1 | LPC 24:0 |
| Indoline | Hydrocortisone | LPE 20:4 sn-1 | SM d18:1/12:0 |
| Dl-Glutamic acid | carnitine C14_3 | LPE 20:4 sn-2 | SM 32:2 |
| D-Pipecolinic acid | Tetradecadiencarnitine | LPE 20_3 | SM 32:1 |
| D-Methionine | Tetradecenoylcarnitine | LPC P-18:1 | SM 33:1 |
| L-Carnitine | Sphingosine-1-phosphate | LPC P-18_0 | PE O-34:3 |
| 5-Formylsalicylic acid | Hexadecenoylcarnitine | LPC O-18:1 | SM 34:2 |
| 5-Methoxysalicylic acid | Palmitoylcarnitine | LPC 17:0 | SM 34:1 |
| D(-)-Arginine | linolenyl carnitine | LPC O-18:0 | SM 34:0 |
| Propylparaben | Linoleyl carnitine | LPC 18:2 sn-1 | PC 30:0 |
| L-Tyrosine | Oleoylcarnitine | LPC 18:2 sn-2 | SM 35:2 |
| Caffeine | Stearoylcarnitine | LPC 18:1 sn-1 | PE 34:2 |
| L-Acetylcarnitine | GCDCA | LPC 18:1 sn-2 | PE O-36:5 |
| D(+)-Tryptophan | GDCA | LPC 18:0 sn-1 | SM 36:3 |
| L-Kynurenine | LPE 16:1 | LPC 18:0 sn-2 | SM 36:2 |
| Butyrylcarnitine | LPE 16:0 | LPE 22:6 sn-1 | PC 32:2 |
| Valerylcarnitine | Glycocholic acid | LPE 22:4 sn-1 | SM 36:1 |
| Hexanoylcarnitine | LPC 14:0 sn-1 | LPC 20:5 | PC 32:1 |
| Phenylacetyl-L-glutamine | LPC 14:0 sn-2 | LPC 20:4 sn-1 | PC 34:0 |
| trans-Vaccenic acid | LPE 18:2 sn-1 | LPC 20:3 sn-1 | PC O-34:2 |
| 2-Octenoylcarnitine | LPE 18:2 sn-2 | LPC 20:2 sn-1 | PC 33:1 |
| Octanoylcarnitine | LPE 18:1 sn-2 | LPC 20:2 sn-2 | PE O-38:7 |
| Arachidonic acid | LPC O-16:1 | LPC 20:1 sn-1 | PE O-38:6 |
| Decadienoylcarnitine | LPE 18:0 sn-1 | LPC 20:1 sn-2 | PC 34:4 |
| Decenoylcarnitine | LPC 15:0 sn-1 | LPC 20:0 sn-1 | PC 34:3 |
| Carnitine C10_1 | LPC O-16:0 | LPC 20:0 sn-2 | PC 16:0/18:2 |
| Decanoylcarnitine | LPC 16:1 sn-1 | LPC 22:6 sn-1 | PC 34:1 |
| carnitine C12:2 | LPC 16:0 sn-1 | LPC 22:5 sn-1 | PE 38:6 |
| Carnitine C12_1 | LPC 16:0 sn-2 | LPC 22:4 | PC O-36:5 |
| FFA 10:0 | 3-Indolepropionic acid | FFA 16:2 | FFA 18:0 |
| Phenyl sulfate | Citric acid | FFA 16:1 | FFA 19:1 |
| Arginine | FFA 12:0 | FFA 16:0 | FFA 19:0 |
| Hippuric acid | Tryptophan | phenylacetylglutamine | FFA 20:5*2 |
| Myoinositol | Indolelactic acid | FFA 17:1 | FFA 20:5*1 |
| Tyrosine | Indoxyl sulfate | FFA 17:0 | FFA 20:4 |
| Hydroxyphenyl lactic acid | FFA 14:1 | FFA 18:4 | FFA 20:3 |
| 4-Pyridoxic acid | FFA 14:0 | FFA 18:3 | FFA 20:2 |
| Indole-3-acrylic acid | FFA 15:0 | FFA 18:2 | FFA 20:1 |
| P-cresol sulfate | FFA 16:3 | FFA 18:1 | Phenylalanylphenylalanine |
| PC O-36:4 | FFA 20:0 | 3-Hydroxybutyrate | Glycochenodeoxycholate |
| PC 35:3 | FFA 22:6 | Creatinine | Glycodeoxycholate |
| PC 35:2 | FFA 22:5 | proline | Glycoursodeoxycholic acid |
| L-Thyroxine | FFA 22:2 | Hexanoic acid | Glycocholate |
| PC 36:6 | FFA 22:1 | 2-Hydroxy-3-methylbutyric acid | LPC16:0 |
| PC 36:5 | FFA 22:0 | 2-Ketohexanoic acid | Taurodeoxycholate |
| PC 36:4 | FFA 24:1 | Malic acid | Taurochenodeoxycholate |
| PC 36:3 | Dehydroepiandrosterone Sulfate | Octanoic acid | Lithocholylglycine 3-sulfate |
| PC 36:2 | FFA 24:0 | Glutamine | Taurocholate |
| PC O-38:6 | Androsterone sulfate-3 | Glutamic acid | LPC18:0 |
| PC O-38:5 | Androsterone sulfate-1 | Oxypurinol | Glycochenodeoxycholic acid 3-sulfate |
| PC 38:6 | Androsterone sulfate-4 | Histidine | Glycodeoxycholic acid 3-sulfate |
| PC 38:5 | Androsterone sulfate-2 | FFA 9:0 | Glycoursodeoxycholic acid 3-sulfate |
| PC 38:4 | Lithocholate | Phenylalanine | Glycochenodeoxycholic acid 3-glucuronide |
| Lactic acid | Ursodeoxycholic acid | Uric acid |  |
